# P13 Growing cases of infective endocarditis following transcatheter aortic valve implantation (TAVI): complex clinical case highlighting the challenges in treating a patient with *Enterococcus faecalis* infection following TAVI procedure

**DOI:** 10.1093/jacamr/dlae217.017

**Published:** 2025-01-23

**Authors:** Sachintha Wijesinghe

**Affiliations:** Guy’s and St Thomas’ NHS Foundation Trust, London, UK

## Abstract

**Background:**

Infective endocarditis (IE) is a rare but a lethal complication following transcatheter aortic valve implantation (TAVI). IE following TAVI occurs in about 0.3–2.0 cases per 100 person-years with most common causative pathogens being enterococci and staphylococci.^1^ Despite the low incidence rate, it is a life-threatening cause of sepsis with a high in-hospital mortality rate and poor long-term prognosis. The risk is significantly higher first-year post TAVI accounting up to 62% of cases^2^. TAVI is a minimally invasive procedure used to treat severe aortic stenosis, alternative to surgery. Enterococcus is recognized as the leading causative pathogen due to the common transfemoral approach in TAVI and groin proximity.^2^ Overall, TAVI-IE is expected to grow exponentially in the coming years with recent rise in TAVI procedures.^1^ This topic was discussed in our IE Network Meeting 2024, held at Guy’s and St Thomas’ Hospital (GSTT) with the same objective, to raise awareness and highlight TAVI-IE management challenges.

**Clinical case:**

An 84-year-old man with background of previous TAVI 7 months ago in his home country, with pacemaker implanted 6 days post-TAVI due to heart block complication. Since then, recurrent admissions to hospital with fever with no clear source of infection. On arrival to the UK, he presented to local emergency department with high-grade fever and hot, swollen knee with history of osteoarthritis. Knee aspirate showed no organism growth but three out of six blood culture bottles grew *Enterococcus faecalis*. Transthoracic echocardiogram revealed no obvious mass or vegetation, but a PET-CT confirmed a small septic focus at proximal pacing lead of pacemaker. CT head for ongoing confusion work-up showed no embolic events. All investigations suggested device related endocarditis. Following the IE MDT, commenced a 6 week course of antibiotics. Initially was started on vancomycin and gentamicin but soon switched to IV ceftriaxone 2 g 12 h and IV amoxicillin 2 g 4 h as standard therapy for *E. faecalis*. The pacemaker was extracted and the patient was discharged on recovery. The patient relapsed 2 weeks later with positive *E. faecalis* blood cultures and MRI brain showing multiple infarcts. A definitive TAVI-related IE diagnosis was made and the patient was deemed not a good surgical candidate in view of his frailty and new cerebrovascular event. He completed another 6 week course of IV ceftriaxone and IV amoxicillin, and this time was discharged with life-long amoxicillin 1 g BD oral as secondary prophylaxis.

**Conclusions:**

TAVI-IE management is challenged by factors including infection severity, drug resistance, comorbidities, frailty and diagnostic delays. General antimicrobial treatment duration lasts at least 6 weeks guided by microbiological profiling and estimated MIC.^2^ Up to 50% develop IE within 100 days of TAVI, and *Enterococcus* species show a high antimicrobial resistance and rate of relapse.^1,2^ According to European Society of Cardiology (ESC) guidelines, peri-operative antibiotic prophylaxis should be considered in high-risk TAVI-IE patients and at least a year post-TAVI. Overall future studies should focus on antibiotic prophylaxis, antibacterial biomaterial for prosthetic devices, diagnostic criteria and novel imaging techniques for TAVI IE to prevent delay in diagnosis and management.^1^
